# Differential Modulation of Local Field Potentials in the Primary and Premotor Cortices during Ipsilateral and Contralateral Reach to Grasp in Macaque Monkeys

**DOI:** 10.1523/JNEUROSCI.1161-23.2024

**Published:** 2024-04-08

**Authors:** Ali Falaki, Stephan Quessy, Numa Dancause

**Affiliations:** ^1^Département de Neurosciences, Faculté de Médecine, Université de Montréal, Montréal, Québec H3C 3J7, Canada; ^2^Center interdisciplinaire de recherche sur le cerveau et l’apprentissage (CIRCA), Université de Montréal, Montréal, Québec H3C 3J7, Canada

**Keywords:** hand movement, laterality, local field potential, motor control, premotor cortex, primary motor cortex

## Abstract

Hand movements are associated with modulations of neuronal activity across several interconnected cortical areas, including the primary motor cortex (M1) and the dorsal and ventral premotor cortices (PMd and PMv). Local field potentials (LFPs) provide a link between neuronal discharges and synaptic inputs. Our current understanding of how LFPs vary in M1, PMd, and PMv during contralateral and ipsilateral movements is incomplete. To help reveal unique features in the pattern of modulations, we simultaneously recorded LFPs in these areas in two macaque monkeys performing reach and grasp movements with either the right or left hand. The greatest effector-dependent differences were seen in M1, at low (≤13 Hz) and γ frequencies. In premotor areas, differences related to hand use were only present in low frequencies. PMv exhibited the greatest increase in low frequencies during instruction cues and the smallest effector-dependent modulation during movement execution. In PMd, δ oscillations were greater during contralateral reach and grasp, and β activity increased during contralateral grasp. In contrast, β oscillations decreased in M1 and PMv. These results suggest that while M1 primarily exhibits effector-specific LFP activity, premotor areas compute more effector-independent aspects of the task requirements, particularly during movement preparation for PMv and production for PMd. The generation of precise hand movements likely relies on the combination of complementary information contained in the unique pattern of neural modulations contained in each cortical area. Accordingly, integrating LFPs from premotor areas and M1 could enhance the performance and robustness of brain-machine interfaces.

## Significance Statement

We compared local field potentials (LFPs) from the primary motor cortex (M1) and the dorsal and ventral premotor cortices (PMd and PMv) while monkeys performed reach and grasp with their contralateral or ipsilateral hand. In general, hand-related differences were greater in M1 than those in premotor areas. During both contralateral and ipsilateral trials, LFPs were more similar when comparing the two premotor areas than when comparing M1 to PMd or PMv. However, the pattern of modulations in each area had unique features. The combination of these signals is likely essential to support the flexibility and complexity of unilateral hand movements. Our results help us to understand the neural substrate that allows the cortical areas to concurrently contribute to the different aspects of movement planning and production.

## Introduction

Performing hand movements is associated with coordinated modulation of neuronal activity across several brain regions, including the primary motor cortex (M1) and the dorsal and the ventral premotor areas (PMd and PMv, respectively; Tanji et al., 1988; Takahashi et al., 2017). Intracortical local field potentials (LFPs) have been used to study brain dynamics and decode the function of motor areas (Buzsaki et al., 2012; Pesaran et al., 2018). Modulations in LFP signals depend on the activity of the local neural population (Denker et al., 2011) and the synaptic inputs into the area (Herreras, 2016) and thus provide complementary information to spiking activity. Previous studies have shown that LFPs in M1, PMd, and PMv are modulated in preparation and during contralateral reach and grasp movements (O’Leary and Hatsopoulos, 2006; Rule et al., 2017). All three areas contain information about the reach and grasp kinematics and kinetics of both proximal and distal muscles involved (Spinks et al., 2008; Hao et al., 2014; Milekovic et al., 2015). Much like for isolated neurons, LFPs in premotor areas also highlight their potential involvement in more cognitive processes such as attention, task structure, or intentions (Mattia et al., 2010; Pani et al., 2014).

While LFPs in M1, PMd, and PMv clearly contain information about contralateral movements, many studies comparing LFPs between M1 and premotor areas have been limited to selective frequency bands (Spinks et al., 2008; Milekovic et al., 2015; Waldert et al., 2015). Studies directly comparing M1 to PMv have suggested they contain similar information about reach and grasp kinematics, although the strength of object-related LFP modulations is greater in M1 while holding the object (Spinks et al., 2008; Bansal et al., 2012) and greater in PMv during movement observation (Waldert et al., 2015). Direct comparison of LFPs in M1 and PMd or between PMd and PMv has been limited to the movement preparation period. Comparing M1 to PMd revealed that both areas are tuned to the intended movement direction (O’Leary and Hatsopoulos, 2006) but that decoding the intended target direction is better when using signals from PMd. Direct comparison of activity in PMd and PMv has shown low correlations between neuron spiking and β oscillations in both areas, suggesting that the dissociation between these two signals is conserved across premotor areas (Rule et al., 2017). Altogether, we thus have a limited understanding of how the patterns of LFP activity differ across M1, PMd, and PMv during contralateral reaching and grasping movements.

In addition to their implication in contralateral movements, a large population of neurons in the premotor cortex (Cisek et al., 2003; Moreau-Debord et al., 2021) and M1 (Ames and Churchland, 2019; Cross et al., 2020) are also modulated during ipsilateral arm movements. Investigations of LFPs during ipsilateral tasks have been mainly limited to M1 and proximal arm control (de Oliveira et al., 2001; Ganguly et al., 2009). They have revealed that LFPs can represent both ipsilateral and contralateral kinematic parameters (Ganguly et al., 2009), though modulation amplitudes are larger during contralateral arm reaches (de Oliveira et al., 2001; Donchin et al., 2001). One study directly compared LFPs in M1 to the supplementary motor area (SMA) and found a greater preference for contralateral arm movements in M1 than in SMA (Donchin et al., 2001). Given that many neurons in PMd and PMv show changes in discharge rate during ipsilateral reach and grasp, it is likely that LFPs are also modulated in these areas during both phases of ipsilateral hand movements. It is however unclear how effector-specific signals in PMd and PMv compare to each other and M1.

To address some of these issues, we recorded the pattern of LFP activity across M1, PMd, and PMv while monkeys performed reach and grasp movements, with either their contralateral or the ipsilateral arm, and analyzed modulations across a relatively large frequency range (0.5–60 Hz). Our results highlight several unique features in the pattern of LFP modulations that occur in each area.

## Materials and Methods

### Subjects

Simultaneous recording of local field potentials and behavioral data was made in two adult, male *Macaca mulatta* monkeys (Monkey Y, 10 kg; Monkey B, 10 kg). Monkeys were housed at the animal facility of the Université de Montréal and brought to the laboratory to conduct the neuronal recording sessions. All experimental and surgical procedures were conducted in compliance with the Canadian Council on Animal Care guidelines and were approved by the Comité de Déontologie de l’Expérimentation sur les Animaux (CDEA) of the Université de Montréal.

### Experimental design and behavioral task trials

**Figure 1.**
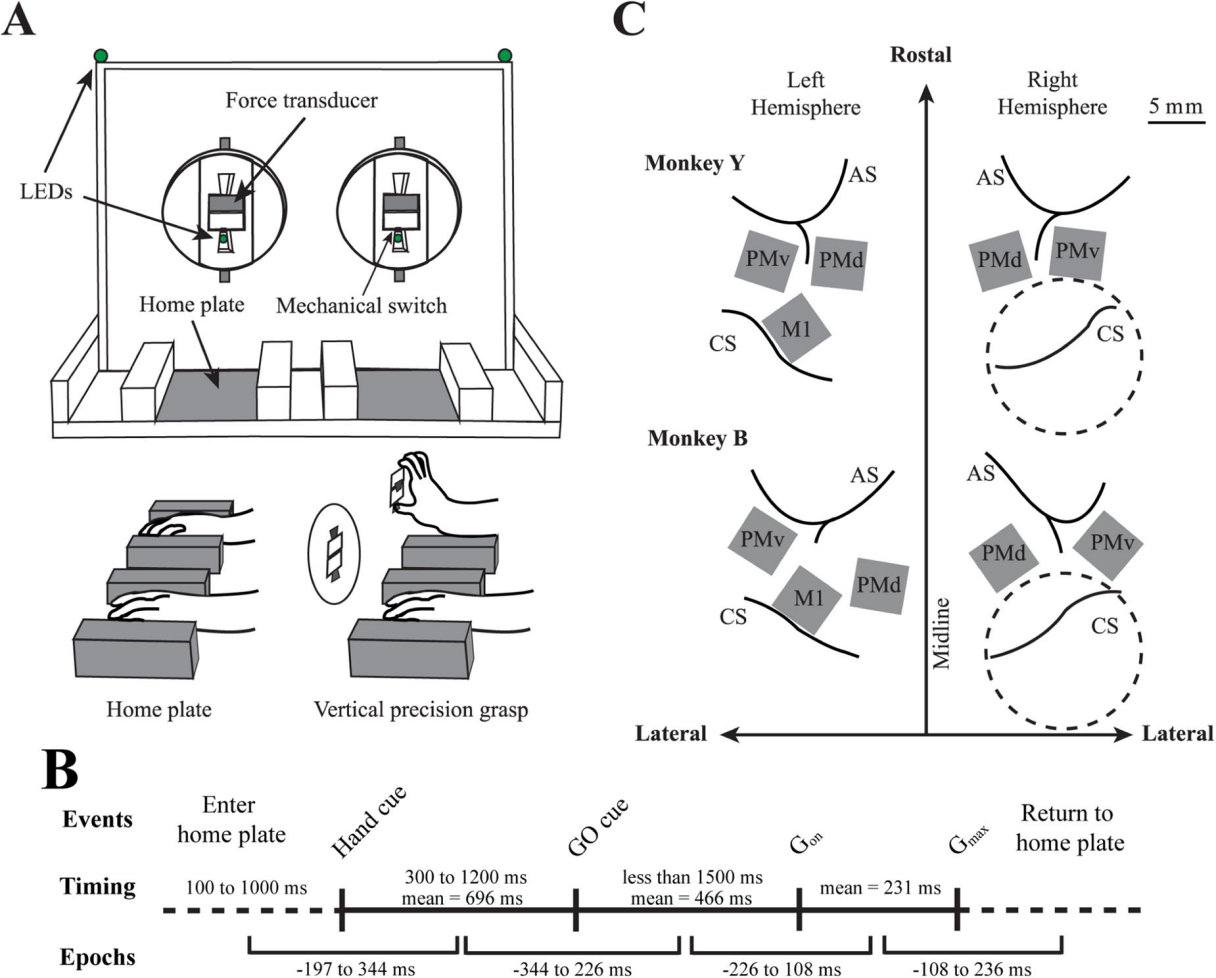
Experimental setup. ***A***, Illustration of the behavioral task. Starting from a home plate position, monkeys had to reach with either the right or the left hand and apply pressure to a force transducer using the thumb and index finger. ***B***, Different phases of the movement and trial events. Each trial was initiated when the monkey placed both hands at the home plates. After a variable delay (Baseline), the trial was initiated by turning on two LEDs, either on the left or the right side of the apparatus, indicating which hand to use (Hand cue). After another variable delay (Preparation), the LEDs were turned off, which provided the GO cue. The monkey then had a maximum of 1.5 s to move its hand from the home plate and reach toward the force transducer (reach). It then had to grasp the force transducer between the thumb and index in a precision grip, apply a force greater than 2.74 N and maintain it for at least 200 ms (grasp). The time of maximal grasp force production (*G*_max_) was used as another behavioral event to align data. If successful, an auditory signal was associated with the juice reward and corresponded to the end of the trial. The monkey could then release the grasp and bring back the hand to the home plate in order to initiate the following trial after a minimum intertrial delay of 1 s. ***C***, Approximate location of the Utah microelectrode arrays and of the chamber (dotted circle) relative to the sulci in the two monkeys. AS, arcuate sulcus; CS, central sulcus; M1, primary motor cortex; PMd, dorsal premotor cortex; PMv, ventral premotor cortex.

Monkeys were trained on a custom-made reach-to-grasp behavioral task. Following instruction cues, they had to reach with either the left or the right arm and apply pressure on strain gauge force transducers using vertical precision grasps. A small mechanical switch was placed on the opposite side of each force transducer to make sure monkeys applied forces with the thumb and index instead of simply pressing down on the force sensor with one digit. While interacting with the task, the monkeys sat ∼28 cm in front of the apparatus in a custom-made primate chair. The force transducers (output compression of 3 mV/V) were placed 9 cm above the start position (home plate), approximately at the elbow level. The chair had removable panels on each side to free the arms and allow the monkeys to interact with the apparatus. On each side of the apparatus, one LED was located at the top corner, and another one was in the slot below each force transducer. Both LEDs were used simultaneously to instruct the monkey which hand to use and when to initiate the movement (i.e., GO cue; Fig. 1*A,B*).

To start a trial, monkeys had to place both hands at the home plate positions. Infrared sensors monitored the presence of the hands during the premovement baseline period. After a variable delay period (100–1,000 ms), the left or right LEDs were turned on to notify the beginning of the trial and which hand to use (hand cue). The other (inactive) hand had to stay at the home plate throughout the trial. After a variable delay of 300 to 1,200 ms, the LEDs were turned off to provide the GO cue. After the GO cue, monkeys had a maximum of 1,500 ms to reach the force transducer, grasp it between the thumb and index (grasp onset; *G*_on_), and apply force levels >2.74 N for at least 200 ms. A continuous auditory signal (1 KHz) informed the monkeys when the force applied reached the threshold. After the 200 ms, the auditory cue changed to 2.5 KHz for 100 ms indicating successful execution of the trial. Successful trials were rewarded with juice (1 ml) delivered with a metal straw fixed in the front of the mouth. The monkey could then release the grip and place the hand back at the home plate to initiate the next trial. The intertrial interval was varied from 1 to 3 s. For each trial, the time of maximal grasp force (*G*_max_) and grasp onset (*G*_on_) were identified offline. For this purpose, the force data were low-pass filtered (20 Hz) using a fourth-order, zero-phase Butterworth filter. For each individual trial, *G*_on_ was defined as the time when the force applied became >1% of the *G*_max_. Figure 1*B* illustrates the time course of trials. It includes the behavioral events that were used for temporal alignment of the neural activity (hand cue, GO cue, *G*_on_, and *G*_max_) as well as the various task epochs around those events. In each session, monkeys performed 25–55 successful precision grasps with each hand, with the order randomized both within and between sessions.

A Tucker-Davis Technologies acquisition system (Tucker-Davis Technologies) using two RZ2 BioAmp processors and custom-made software was used to control the task and collect behavioral data sampled at 5 kHz, including the forces applied by the monkeys and the timing of the hand cue and the GO cue. All behavioral trials were also video recorded at 30 frames per second using a computer webcam located on the left side of the animal at the shoulder level allowing for offline verification.

### Surgical procedures

All surgical procedures were conducted under standard sterile conditions. Once the monkeys achieved a task performance of at least 90% success, they underwent implantation of arrays for chronic neural recordings. Anesthesia was induced by intramuscular injection of ketamine hydrochloride (Ketaset; 15 mg/kg; Pfizer) and maintained using isoflurane (∼2–3% in 100% O_2_; furane; Baxter) after tracheal intubation. An intravenous catheter was inserted in the saphenous vein to infuse Ringer's lactate solution (10 ml/kg/h) and administer drugs intravenously during the surgery. The head was shaved and washed with antimicrobial skin cleaner (Germi-Stat, 4%; chlorhexidine gluconate, 4% w/v; Ceva Animal Health) and povidone–iodine solution (70% betadine and 30% isopropyl alcohol). Before the surgery, monkeys received a subcutaneous injection of buprenorphine (Temgesic; 5 µg/kg; Schering-Plough) and an intramuscular injection of carprofen (4 mg/kg; Rimadyl, Zoetis Canada) to control postsurgical pain and inflammation, respectively. Also, intramuscular injections of atropine (atropine sulfide, 0.04 mg/kg; Rafter 8 Products) and dexamethasone 2 (Dexacort 2, 0.5 mg/kg; Rafter 8 Products) were given to avert salivation and swelling of the brain, respectively. To further prevent swelling, a dose of mannitol 20% (1,500 mg/kg; Fresenius Kabi Canada) was slowly administered intravenously at the beginning of the craniotomy. Throughout the surgery, vital signs (respiratory CO_2_, electrocardiogram, and arterial oxygen saturation) were monitored while the body temperature was kept at 36.5–37°C with a self-regulated heating blanket (Harvard Apparatus).

Array implantations were done according to the guidelines published by Blackrock Neurotech. The monkeys were positioned in a stereotaxic frame, and the frontal cortex of both hemispheres was exposed by craniotomy and durectomy. Each monkey was implanted with five Utah microelectrode arrays (4.2 × 4.4 mm; 96 1.5 mm electrodes; Blackrock Neurotech). Arrays were positioned to simultaneously record neural data from the forelimb representation in the left M1 and in PMd and PMv of both hemispheres (Fig. 1*C*). The precise locations of array implantations were determined based on sulcal landmarks (arcuate and central sulci; Fig. 1*C*). Few exploratory cortical sites were also tested using short intracortical microstimulation (ICMS) trains (i.e., trains delivered at 1 Hz, each train consisting of 13 monophasic cathodal pulses of 0.2 ms duration delivered at 350 Hz) using a maximum current intensity of 60 µA (Dancause et al., 2008). The isoflurane was turned off, and animals were transitioned to ketamine–diazepam sedation (Ketaset, ∼10 mg/kg/h; Valium, ∼0.01 mg/kg/h). A glass-coated tungsten microelectrode (impedance, ∼0.5 MΩ; FHC) mounted on a micromanipulator (David Kopf Instruments, model 2662) was lowered perpendicular to the cortex at each site. During these short procedures, we located the border between the hand and the face representations in M1 and implanted the array medially to sites evoking orofacial responses. In the premotor cortex, we stimulated areas lateral (PMv) and medial (PMd) to the arcuate spur until we found one site evoking distal movements in each area and implanted the arrays over these sites. In Monkey B, however, the placement of the PMd array in the left hemisphere was also affected by a large blood vessel that crossed the region mediolaterally. The array was implanted immediately caudal to that large vessel.

We kept the dura covering the central sulcus of the right hemisphere intact and implanted a 20-mm-diameter cylindrical chamber made of polyether ether ketone over it. The chamber provided access to the hand representation of the right M1 and primary somatosensory (S1) cortex required in a subsequent series of experiments. Once the arrays were implanted, the brain was covered with DuraGen (DuraGen Plus; Integra LifeSciences) and bone cement (Palacos; Heraeus Medical Components). The connectors were secured in an acrylic box placed on top of the head, rostrally to a head fixation post. The entire assembly (the acrylic box with connectors, the wires, the chamber, and the post) was fixed to the skull using anchoring surgical screws and bone cement.

At the end of the procedure, a dose of Baytril (enrofloxacin, 5 mg/kg; Bayer) was injected intramuscularly to prevent infection. Postoperative care included additional doses of Baytril and carprofen given orally for 2 d, as well as gabapentin (10 mg/kg; Pfizer) given twice a day for 3 d following the surgery.

### Identification of the physiological location of the implanted arrays

To better define the physiological location of the arrays, we analyzed both neuronal spiking activity and the movements evoked with short ICMS trains using the electrodes of the arrays in the left M1 in both monkeys and left PMd in Monkey Y. For neuronal spiking, we used the peak discharge rate to characterize the activity of neurons (Moreau-Debord et al., 2021). To do so, the mean firing rates were computed within epochs around the hand cue, GO signal, and grasp onset. These rates were then normalized to the baseline (400–100 ms prior to the hand cue). For each neuron, we identified the task epoch with peak discharge rate, independently of the arm used (i.e., only one count per neuron). Then, for each monkey and cortical area, we calculated the proportion of recorded neurons having their peak discharge rate in each task epoch. In general, in each array, we found neurons modulated during each epoch of the trials (Table 1). Across monkeys, 75.9% of the neurons recorded in M1 had their peak discharge rate during grasp. In PMd and PMv, these neurons accounted for 33.3 and 36.4% of the recorded neurons, respectively. Notably, the array with the most pronounced bias was the one in the left M1 of Monkey Y, where 87.3% of the recorded neurons displayed their peak activity during the grasp epoch. However, even for that array, there were also neurons with peak discharge rates during the hand cue and the reach epochs.

**Table 1.** Characteristics of neural spiking activity at different task epochs

	Area	Number of single units	Single units with their peak discharge rate during different task epochs [*N* (%)]		
			Hand cue	GO cue	Grasp onset
Monkey Y	Left M1	63	6 (9.5)	2 (3.2)	55 (87.3)
	Left PMd	39	11 (28.2)	8 (20.5)	20 (51.3)
	Right PMd	22	6 (27.3)	6 (27.3)	10 (45.5)
	Left PMv	12	4 (33.3)	5 (41.7)	3 (25)
	Right PMv	23	9 (39.1)	4 (17.4)	10 (43.5)
Monkey B	Left M1	16	8 (50)	3 (18.8)	5 (31.2)
	Left PMd	–	–	–	–
	Right PMd	76	39 (51.3)	17 (22.4)	20 (26.3)
	Left PMv	45	21 (46.7)	7 (15.5)	17 (37.8)
	Right PMv	22	16 (72.8)	2 (9)	4 (18.2)

For each monkey and cortical area, the preferred task epoch of each single unit was identified by determining the epoch in which the neuron had its maximal discharge rate, normalized by its average firing rate during baseline (400 to 100 ms prior to the hand cue). Trials performed with either hand are pooled. Each neuron has only one count that includes both the task epochs and the hand used.

Approximately 6 months after the array implantation, ICMS motor mapping using short trains delivered through three arrays was done during a few experimental sessions. These data were collected for another set of experiments that have been previously published (Bonizzato et al., 2023). During these sessions, monkeys sat calmly while treats were intermittently offered between blocks of stimulations. Stimulation trains were delivered (10–20 repetitions) from each electrode at a frequency of 1 Hz. M1 stimulations were delivered with a current intensity of 30 µA in Monkey Y and 40 µA in Monkey B. Note that the intention in these data collections was to use a single stimulation intensity that was high enough to evoke clear movements across several electrodes. For each electrode, when a response was evoked, the intensity was lowered to identify the movement evoked at threshold current intensity as in previous studies (Dancause et al., 2006b; Touvykine et al., 2016). In Monkey Y, stimulations evoked responses in 87 electrodes: 58 in hand muscles (66.7%) and 29 in forearm muscles (33.3%). In Monkey B, stimulations evoked responses in 29 electrodes: 20 in hand muscles (69.0%) and 9 in forearm muscles (31.0%). For premotor areas, only the left PMd of Monkey Y was stimulated with 100 µA. Stimulations evoked responses in 54 electrodes: 29 in hand muscles (53.7%) and 25 in forearm muscles (46.3%).

Together, these findings indicate that the arrays were implanted in regions of M1 and the premotor cortex involved in the control of reach and grasp. We were able to gather reasonable complementary physiological information for all arrays, except for the one in the left PMd of Monkey B. While we could clearly hear neural hash in the background on several channels, we could not convincingly isolate signals from individual neurons. As a control, we compared the pattern of modulations recorded from this array to the one in the opposite PMd in this monkey and found the two consistent. We thus decided to keep data from this array in our analyses.

### Neural recording

Each array was connected to three Omnetics connectors; each collecting data from 32 electrodes. Out of ten implanted arrays, eight had platinum (Pt) electrodes coated with parylene C, and the other two arrays had iridium oxide (IrOX) electrodes. According to the impedance worksheet from the company, Pt electrodes had a working impedance of 301 ± 84 KΩ (min, 102 KΩ; max, 695 KΩ). IrOX electrodes had an impedance of 55 ± 4 KΩ (min, 43 KΩ; max, 67 KΩ). Any Pt electrode with a tested impedance of >800 KΩ in the worksheet was considered dysfunctional and removed from data analysis. Consequently, the data from 11 electrodes were discarded. The IrOX arrays were implanted in Monkey B within the left M1 and the right PMd.

Two RZ2 BioAmp processors along with two 128-channel preamplifiers (PZ2-128) were used to collect simultaneous neuronal activity (intracortical LFPs and neuronal spiking activity). Our equipment supported a maximum number of 256 recording electrodes (by means of eight connectors each collecting data from 32 electrodes). Thus, different brain areas were selected in various recording sessions. This method enabled us to have several recordings of each implanted area across recording sessions. For the current study, we selected six sessions for Monkey Y (starting 9 d after the surgery) and five sessions for Monkey B (starting 51 d after the surgery), over a period of 2 weeks, to gather sufficient data from M1, PMd, and PMv.

LFP data with a frequency below 500 Hz were recorded continuously with a sampling frequency of 2 kHz. Simultaneous spiking activities from the same 256 electrodes were bandpass filtered (100–5 kHz), digitized, and recorded at 24.5 kHz. Spikes were detected automatically by the Tucker-Davis Technologies system using an automatic threshold crossing method (4× baseline noise standard deviation; SD). Electrode-specific thresholds were visually inspected at the beginning of the experiment and manually modified if needed and then were locked in place for the recording session. Whenever a threshold crossing was detected by the data acquisition system, a time interval of 1,228 µs (30-sample spike waveform) of the neural activity was recorded. Plexon Offline Sorter (Plexon) was used to sort recorded suprathreshold waveforms offline using principal component analyses. Single units were identified by their clearly separated clusters in comparison with other recorded signals. Sorted spike times for each electrode were used to despike LFPs using linear interpolation (see below, Neural data preprocessing and trial rejection).

### Neural data preprocessing and trial rejection

Neural data were analyzed using custom software written in MATLAB R2018b (MathWorks) and Brainstorm (Tadel et al., 2011; http://neuroimage.usc.edu/brainstorm). For both PMd and PMv, neural data from the left and right hemispheres were combined and classified based on the hand performing the task (i.e., ipsilateral or contralateral hand in relation to the array). Brainstorm was used to preprocess and clean LFPs. Raw LFP data were first bandpass (0.5–500 Hz, zero-phase) and notch filtered (60 Hz and its harmonics up to 300 Hz). Filtered LFPs were visually inspected to identify the presence of significant noise and artifacts in specific electrodes (e.g., broken electrodes), which were excluded. Such artifacts were easily detected by their high voltage values, well beyond the typical physiological range of cortically recorded LFPs (±400 µV). Data from the remaining electrodes were then individually inspected to remove specific trials containing noise or artifacts. Trials during which the filtered LFP exceeded five times the SD of the filtered data at any time between −2 and +2 s interval around the GO cue were considered noisy and excluded from further analyses. At the end of this first cleaning procedure, the data set needed to include at least 15 trials with each hand to be kept for further analyses.

Independent component analysis (Bell and Sejnowski, 1995; Whitmore and Lin, 2016) was applied to LFPs (rows of data) collected by each individual connector (32 electrodes minus those excluded in previous steps). Independent components were visually inspected, and any component associated with artifacts (e.g., chewing, lip, or head movements) or volume-conducted noise was removed. Volume-conducted noise was identified based on its distinct pattern of activity and uniform manifestation across many electrodes. These common activity patterns, presumably resulting from strong distant sources, were not coupled with the preparation or execution of the reach and grasp. Reconstructed LFPs were visually inspected one more time to exclude any possible noisy electrode or trial from the analysis. Finally, to decrease the potential effects of spiking activity, spike-related information (spiking time and the corresponding electrode) was used to remove a 3 ms window (from 1 ms before to 2 ms after the recorded spike time) and replace it with linearly interpolated data points (Waldert et al., 2013).

### Production of LFP spectrograms

After data preprocessing, spectral analysis was used to characterize the frequency structure of LFP activity up to 60 Hz using the multitaper time–frequency analysis embedded in Brainstorm. We focused on signals below 60 Hz since high-frequency LFP activity (e.g., high-γ activity; >80 Hz) can easily be contaminated by spiking activity (Ray and Maunsell, 2011; Zanos et al., 2011; Waldert et al., 2013). The multitaper technique estimates the frequency structure from multiple orthogonal tapers (Jarvis and Mitra, 2001). It allows a more accurate estimation of the LFP spectrum by providing better control of time and frequency smoothing and by reducing the leakage across frequency bins (van Vugt et al., 2007).

**Figure 2.**
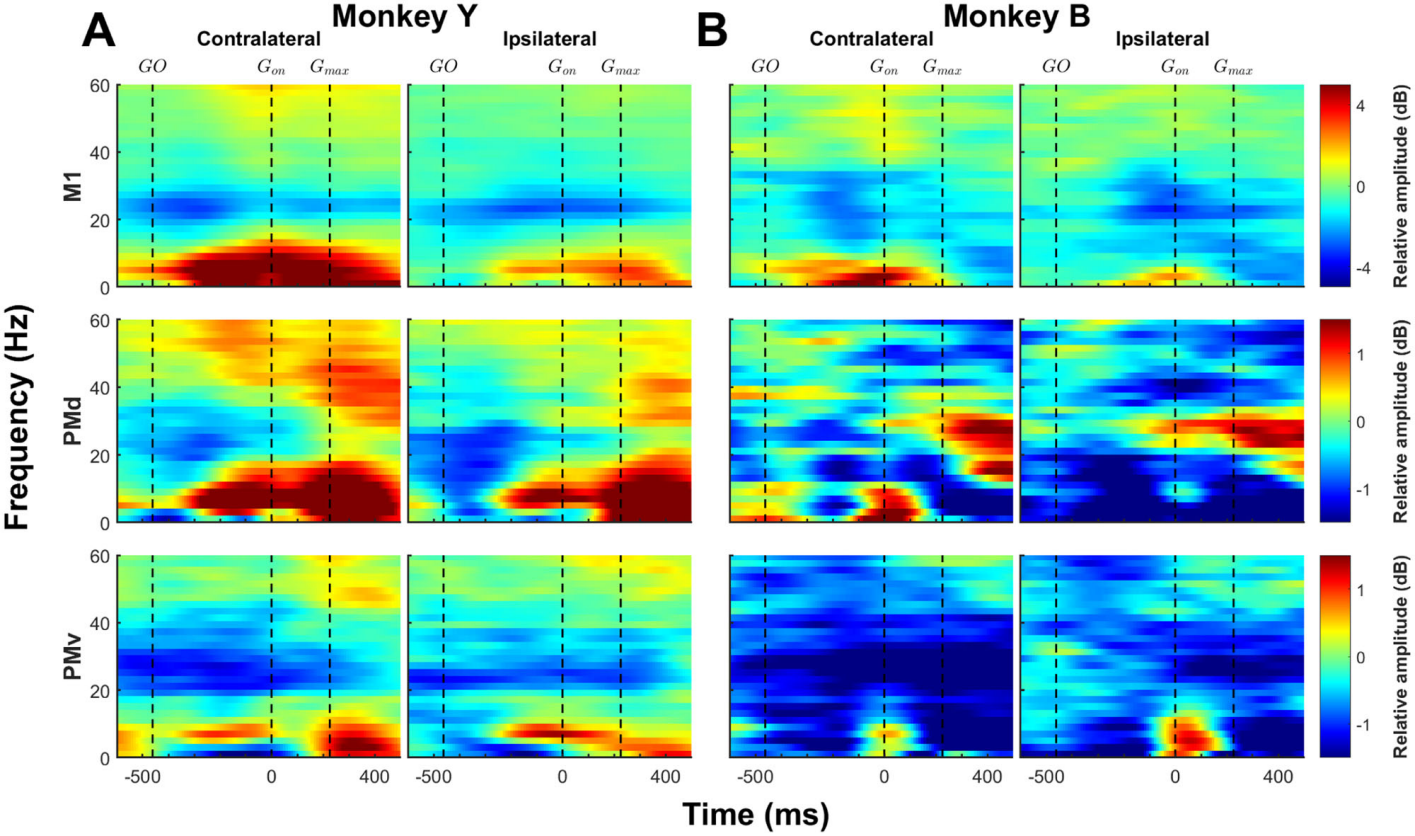
LFP spectrograms of individual monkeys during movements of the contralateral and ipsilateral hand. ***A***, Spectral modulations of LFPs recorded in M1 (top row), PMd (middle row), and PMv (bottom row) when Monkey Y performed the task with the contralateral hand (left column) or the ipsilateral hand (right column). ***B***, Spectral modulations of LFPs recorded in Monkey ***B***. In both panels, LFP data are aligned to grasp onset (*G*_on_) and extends from −600 to 500 ms around this event. The median time values for the GO cue and the time at which monkeys exerted maximal grasp forces (*G*_max_), relative to *G*_on_ are marked by vertical dashed lines. For each monkey and brain area, spectrograms were scaled similarly for contralateral and ipsilateral trials. Although each cortical area showed a unique pattern of modulations, each cortical area tended to have a similar pattern in both in Monkey Y and Monkey B. Data in the two animals were thus merged for subsequent analyses.

**Figure 3.**
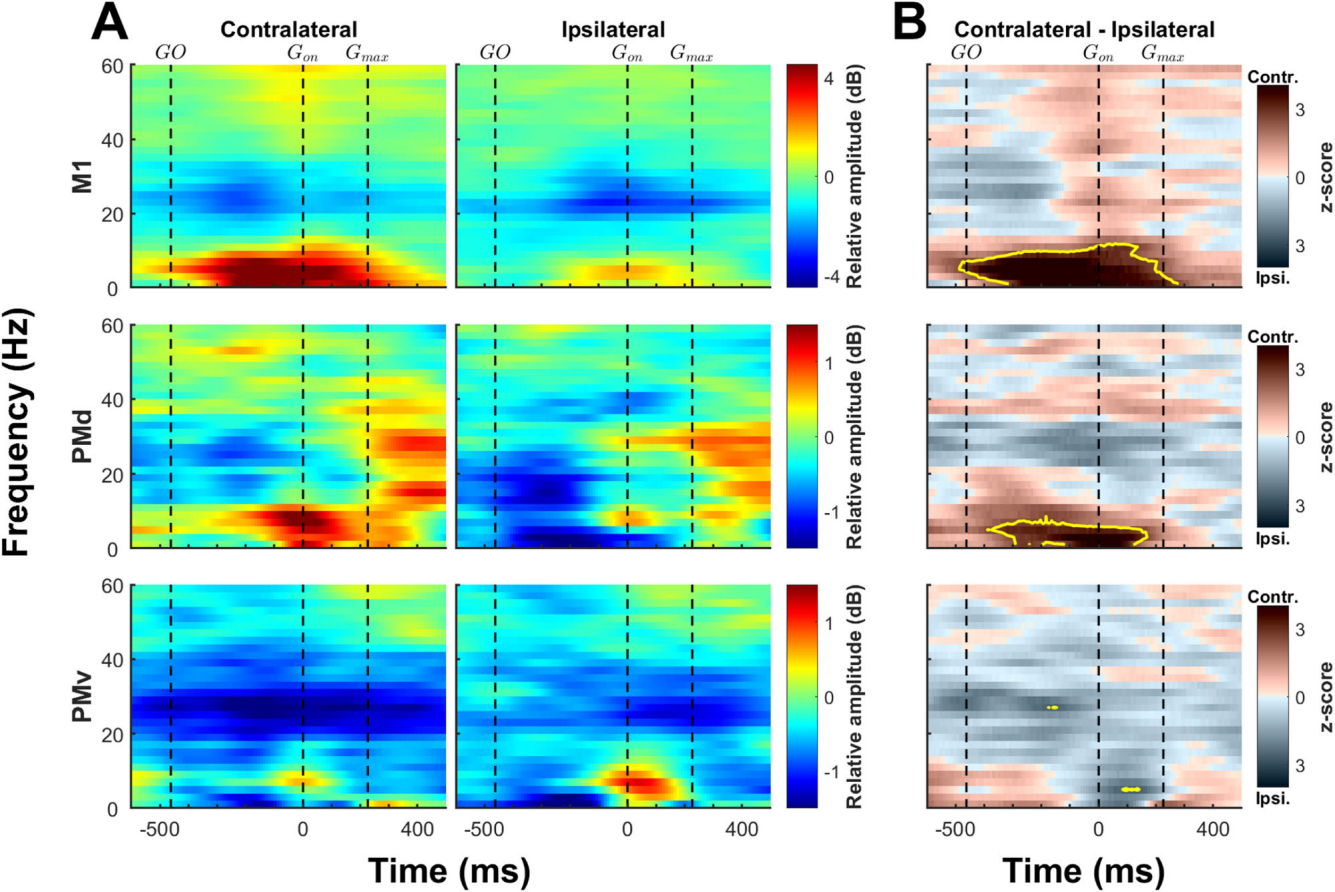
LFP spectrograms during movements of the contralateral and ipsilateral hand. ***A***, Spectral modulations of LFPs recorded in M1 (top row), PMd (middle row), and PMv (bottom row) while the money performed a reach-to-grasp task with the contralateral hand (left column) or the ipsilateral hand (right column). Data is aligned to grasp onset (*G*_on_; vertical dashed lines). In addition to *G*_on_, the median time values for the GO cue and for the time the monkeys exerted maximal grasp forces (*G*_max_) are also indicated by vertical dashed lines. The scale of the relative amplitude of the signal is optimized for each brain area but kept constant for contralateral and ipsilateral trials. In general, amplitudes were much greater in M1 than in PMd and PMv. For all three brain areas, spectral modulations of LFPs during movements of the ipsilateral hand tended to follow the pattern observed for the movement of the contralateral hand. ***B***, The LFP spectrum plots were subtracted between trials executed with the contralateral and ipsilateral hand. Values close to 0 (indicated by a lighter color) represent frequencies where there is minimal or no difference between the two trial types. The darker shades of brown and gray emphasize frequencies exhibiting higher LFP power during contralateral and ipsilateral trials, respectively. The significant differences are indicated by yellow contours (*p* < 0.01; see Materials and Methods). These plots highlight that LFP modulations exhibited a tendency to be more pronounced during contralateral hand movements in both M1 and PMd, especially in the low frequency range (≤13 Hz). In both areas, significant modulations within the δ and θ and α ranges started at or shortly after the GO cue continuing through the reach movement and ending into the grasp epoch. In contrast, modulations in PMv tended to be similar during movements of either hand.

LFP spectrograms for each monkey (Fig. 2) were computed from data aligned to *G*_on_ behavioral event using a 500 ms Hann taper shifted in 10 ms steps resulting in a 2 Hz frequency resolution. This simultaneously brought a high temporal resolution and a high level of frequency detail. For each electrode, the mean across trials was calculated. The averaged spectrums were normalized to the baseline activity (−300 to −100 ms prior to hand cue) and converted to decibels (i.e., the logarithm of the spectrum normalized to baseline; Waldert et al., 2013). We calculated the mean across electrodes from the same brain area, and across sessions for each monkey. Finally, these averaged data per area from each monkey were combined (Fig. 3*A*).

**Figure 4.**
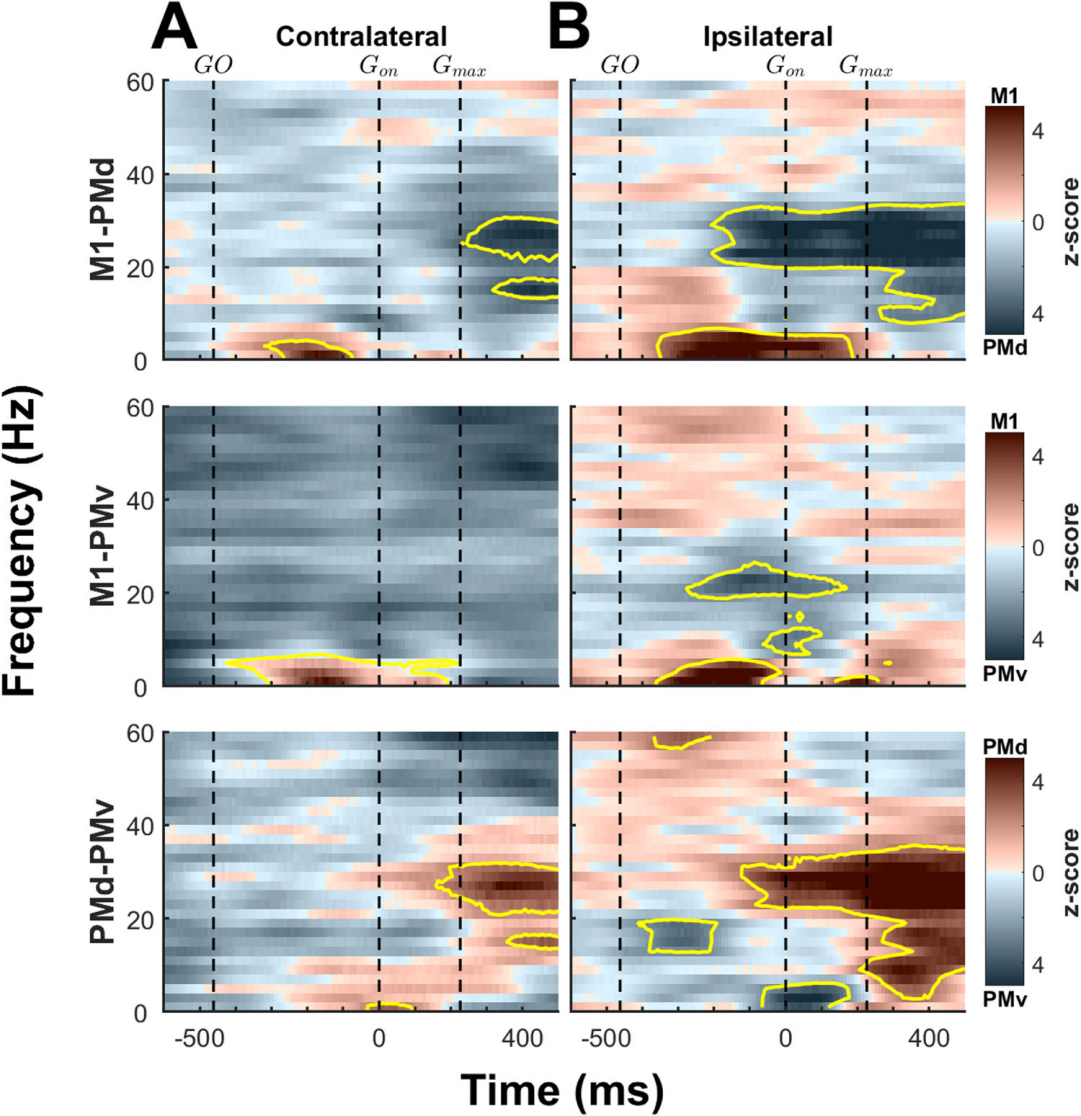
Spectrogram subtraction plots comparing LFP modulation in different cortical areas. Subtraction of the LFP spectrum plots between different brain areas, separated for trials using the contralateral (***A***) and ipsilateral (***B***) hand. The top row shows the differences in modulations between M1 and PMd, the middle row between M1 and PMv, and the bottom row between PMd and PMv. Signals are aligned on grasp onset (*G*_on_) and extend from −600 to 500 ms around *G*_on_. Before subtracting LFP spectrums, values for each brain area were normalized to maximal absolute intensity values. As a result, all plots have the same intensity scaling (color scale on the right). The median timing of the GO cue, *G*_on_, and maximal grasp force (*G*_max_) are shown with dashed vertical lines. Yellow contours emphasize zones with significant differences (*p* < 0.01). Based on this analysis, M1 exhibited greater relative power in low frequencies during reaching compared with PMd and PMv, evident in both contra- and ipsilateral trials. PMd exhibited higher relative power compared with M1 and PMv in frequencies within the β band, both during and after grasp, across both contralateral and ipsilateral trials. In the same frequency band, PMv also showed higher relative power than M1 specifically around grasp onset, but this distinction was observed solely during ipsilateral trials. Furthermore, PMv displayed higher relative power than PMd during grasp onset in the δ band and during reach in the β band, although this difference was noticeable exclusively during ipsilateral trials.

To compare the pattern of modulation between contralateral and ipsilateral trials (Fig. 3*B*), we subtracted ipsilateral from the contralateral LFP spectrogram. To explore the differences in the LFP spectrum between brain regions (Fig. 4), we wanted to highlight when the greater changes in one area occurred in relation to the greater changes in the other area. Because the range of amplitude modulations was much greater in M1 than premotor areas (Fig. 3*A*), we thus scaled each spectrogram to a −1 to 1 range prior to subtraction.

### Amplitude modulations across specific frequency bands

To investigate frequency-specific modulations of the LFP power during different epochs of the task, we separated the signal into four different bands: δ (0.5–5 Hz); θ and α (5–13 Hz); β (15–30 Hz); and γ (30–60 Hz; Newson and Thiagarajan, 2019). For these analyses, the frequency-specific amplitude modulations were generally computed similarly to spectrogram plots. However, to limit the effects of trial duration variability when performing averaged across trials analyses, LFPs of each trial were cut into 4-s-long windows centered on the behavioral events (hand cue, GO cue, *G*_on_, and *G*_max_). This resulted in four partially overlapping segments for each trial. Amplitude modulations were computed for each electrode at different frequency bands in these segments and were normalized to baseline amplitude. Data for each segment were then averaged across electrodes from the same brain area for each frequency band. Finally, we obtained the mean LFP amplitude modulations by averaging the data across sessions and monkeys. For each brain area, this approach resulted in a total of 4 × 4 × 2 (events × frequency band × hand used) segments of neural activity. Since amplitude modulations were obtained from multiple recordings, Gaussian error propagation method was used to propagate the uncertainties of connectors and arrays into the standard error of the mean (SEM) of averaged amplitude modulations. Since probable covariance and noise correlations were neglected, actual SEMs might be larger.

**Figure 5.**
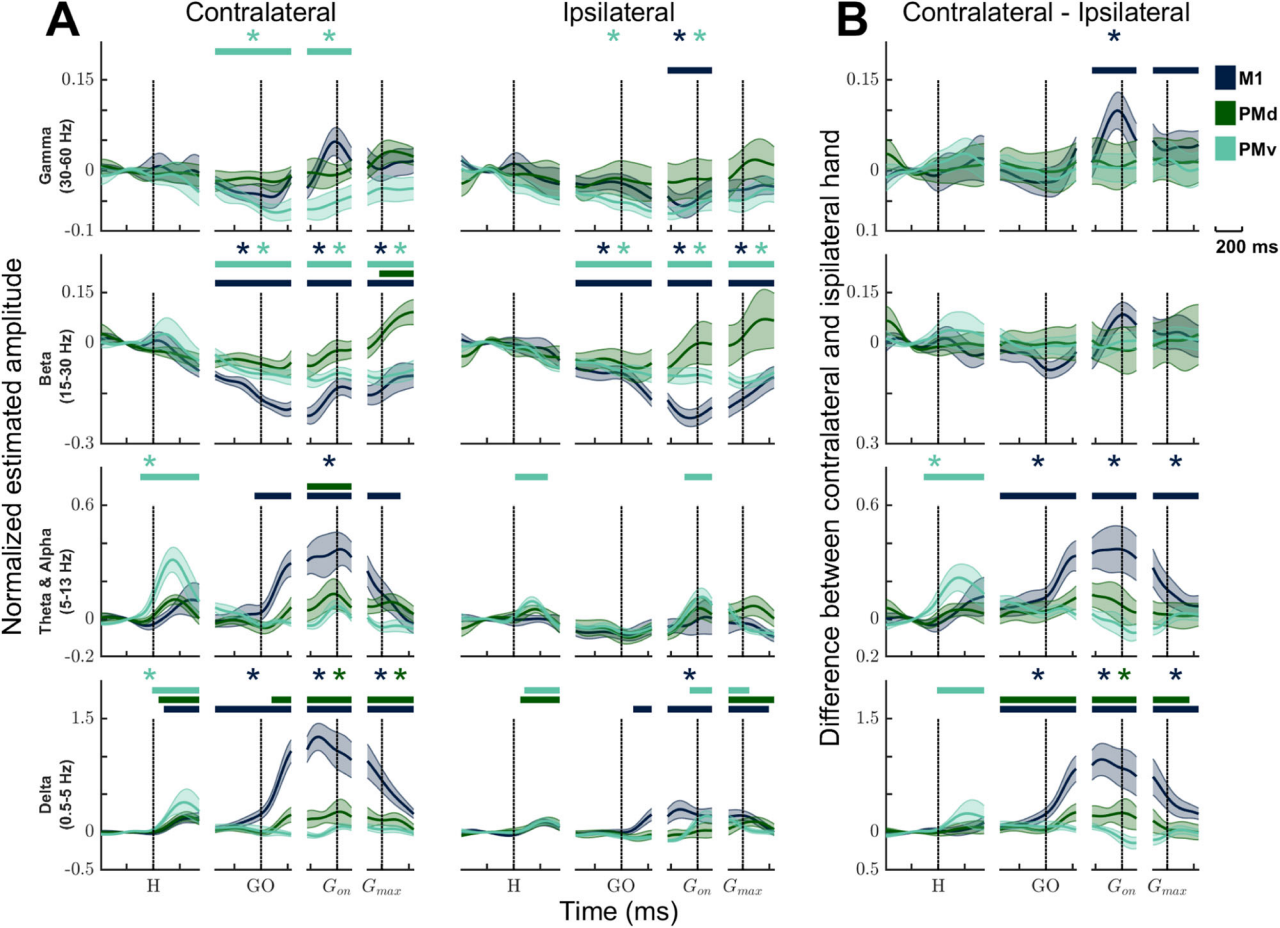
Decomposition of LFP amplitude modulation across frequency bands during movements of the contralateral and ipsilateral hand. ***A***, Mean LFP amplitude modulations separated for δ, θ and α, β, and γ bands for M1 (dark blue), PMd (dark green), and PMv (light green). The left column displays the activity during movements of the contralateral hand while the right column shows the activity during movements of the ipsilateral hand. The shaded area represents the standard error of the mean (SEM). Data were fractionated along trial duration (*x*-axis) to allow alignment to behavioral events (H, hand cue; GO, GO cue; *G*_on_, grasp onset; *G*_max_, maximal grasp). The vertical dotted lines illustrate the time of these different events. The positive and negative values respectively indicate increases and decreases in amplitude modulation relative to the baseline. The horizontal bars at the top of panels indicate intervals when the activity deviated from the baseline by >10 standard deviations (SD), and the difference remained above 2 SD of the mean baseline activity for at least 125 ms, as detailed in the methods. Each cortical area exhibited a distinct modulation pattern across all frequency bands. For δ and θ and α bands, modulations were generally more prominent during trials involving the contralateral hand. Conversely, in the β and γ range, modulations showed lower amplitude and were relatively similar for movements of either hand. In a subsequent analysis, we used one-way ANOVAs with repeated measures to compare the mean LFP activity during each epoch to a baseline period (400 to 100 ms prior to the Hand cue). This analysis was conducted separately for each frequency band. The asterisks positioned at the top of the panels indicate epochs where the mean LFP activity in a specific frequency band significantly differed from the mean activity during the baseline. ***B***, Subtraction plots highlighting the difference between trials involving the contralateral hand compared with the ipsilateral hand. The LFP modulation curves from ipsilateral trials were subtracted from the ones during contralateral trials. Therefore, values above 0 highlight moments during the trial when the LFP power was greater during contralateral hand movements. The values below 0 highlight moments when the LFP power was greater during ipsilateral hand movements. The horizontal bars at the top of the panels signify intervals when the activity from the two hands diverged by >10, SD, and the difference remained above 2 SD of the mean baseline activity for at least 125 ms. In M1, LFP power was greater during contralateral hand trials, a pattern observed across all frequency ranges except for β. In PMd, LFP power showed a higher magnitude during contralateral hand movements specifically in the δ range. Conversely, in PMv, the activity in the δ and θ and α frequencies was notably elevated early in the trial, when the monkeys received instructions to use the contralateral hand. To further investigate the influence of the hand used, we performed an ANOVA to compare the mean LFP activity during movements of either hand for each epoch and each area. This analysis was carried out independently for each frequency band. The asterisks at the top of the panels indicate epochs where the mean LFP activity was significantly different depending on the hand used during the trials.

**Figure 6.**
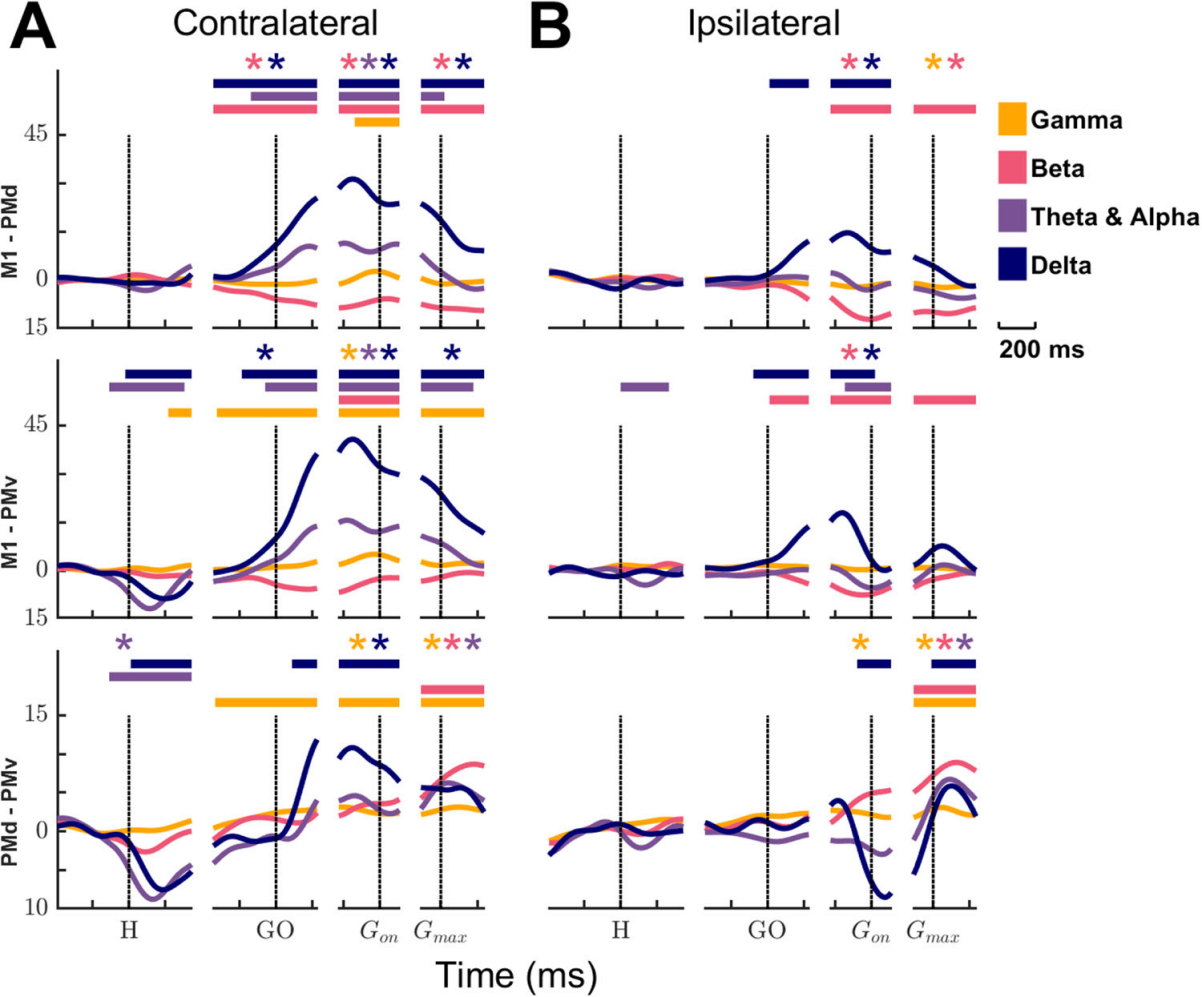
Contrasting frequencies and events specific LFP differences between cortical areas. Differences in amplitude modulations between cortical areas are illustrated for δ (navy), θ and α (violet), β (pink), and γ (yellow). Trials performed with ipsilateral (***A***) and contralateral (***B***) hand are presented separately. As in Figure 5, the data were segmented along the trial duration (*x*-axis) to enhance alignment around behavioral events. The vertical dotted lines denote various task events (H, hand cue; GO, GO cue; *G*_on_, grasp onset; *G*_max_, maximal grasp). The three rows compare LFP amplitude modulations between M1 and PMd (top row), M1 and PMv (middle row), and PMd and PMv (bottom row). Each curve represents the comparison for one frequency band. Values above or below the zero line indicate which of the two compared areas exhibited stronger modulation in that frequency band. Values close to 0 suggest similar activity in the two areas. The horizontal bars at the top of panels indicate intervals when LFP amplitude modulations between the two areas differed by >10 SD, and the difference remained above 2 SD of the mean baseline activity for at least 125 ms. As a secondary analysis, we performed an ANOVA compare for each epoch and each frequency band, the mean LFP activity of the different areas. The asterisks at the top of the panels indicate epochs where the mean LFP activity in a specific frequency band was significantly different for the two compared areas. The plots highlight that differences between M1 and premotor areas were more pronounced during contralateral hand trials compared with ipsilateral trials. Differences between PMd and PMv during contralateral trials were smaller than with M1 and of similar amplitude during contralateral and ipsilateral trials. Nevertheless, there were many moments in trials during which the pattern of activity was substantially different across brain areas. This was true for all frequency bands and for both contralateral and ipsilateral trials. Together, the data indicate that each area displayed a distinct pattern of modulation during reaching and grasping with both contralateral and ipsilateral hands.

Finally, overlapping segments were cut at the averaged midpoint between consecutive events. A gap was inserted at these midpoints to highlight that amplitude modulations were obtained for each epoch separately (Fig. 5). Since the duration of each epoch is based on behavioral events, it varies depending on the hand used or which monkey is doing the task. To ensure consistent trace length for all curves within each epoch, the segmentation was done using the midpoint of the shortest of the four (hand used × monkey) durations (Table 3). To visualize the contrast in amplitude modulations between cortical areas, we calculated the normalized difference for each frequency band (Fig. 6), defined as the difference between the amplitude modulation of two brain areas normalized by the total amplitude modulation within these two areas:

$$\begin{array}{rl} & \mathrm{Normalized}\;\mathrm{difference}\\ & \;=\frac{\text{Area 1 amplitude modulation}-\text{Area 2 amplitude modulation}}{\text{Area 1 amplitude modulation}+\text{Area 2 amplitude modulation}} \end{array}$$

### Statistical analysis and quantification of substantial differences in the pattern of modulations

To identify significant differences in both subtraction spectrograms (Figs. 3*B*, 4), we created a predictor by repetitively selecting (with replacement) data points from the normalized spectrogram of the two data sets (i.e., either the trial type for Fig. 3*B* or two brain areas for Fig. 4) along the time axis. The “shuffled” matrices were then subtracted from each other. This process was repeated 1,000 times. The mean and standard deviation of these differences was then used as the predictor to calculate the *z*-score, serving as a metric for evaluating the significance of the LFP spectrum difference observed in the subtraction plot.

For the analyses of LFP changes in each frequency band (Figs. 5, 6), we used two quantification methods. First, we performed ANOVAs (SAS 9.4; SAS Institute). A one-way ANOVA with repeated measures was applied to identify significant differences in LFP activity within each brain area across various epochs (four levels: H cue, GO, *G*_on_, and *G*_max_) compared with the baseline activity for each frequency band (Fig. 5*A*). A similar test was used to compare the LFP activity within each brain area during contralateral or the ipsilateral trials (Fig. 5*B*). To compare the amplitude of modulation between two brain areas at corresponding epochs, two-way ANOVAs with factors epoch (four levels) and area (two levels: Area 1 and Area 2) were used (Fig. 6). These tests were performed using the averaged amplitude within each epoch and frequency band for each experimental session. Prior to applying all ANOVAs, data were checked for normality assumptions, and if necessary, Box–Cox transformation was used to achieve a normal distribution. When exploring significant effects, we used Bonferroni-corrected *p*-values to account for multiple comparisons (SAS 9.4; SAS Institute). A significance level criterion of *p* < 0.05 was adopted for all statistical comparisons.

However, when the LFP power varied rapidly, averaging the LFP power during the epoch can give false negative findings. For example, θ and α frequencies in M1 rapidly increased after the GO signal (Fig. 5*A*). This change is not detected by the ANOVA. To increase our capacity to highlight substantial LFP modulation of short duration, we used a second quantitative approach consisting of a two-step process that considered both the amplitude and duration of the modulation (Sato and Schall, 2003; Cisek and Kalaska, 2005). To identify when during contralateral or ipsilateral trials each area showed substantial changes of activity in comparison with its baseline (Fig. 5*A*), we calculated the mean and SD of an amplitude modulation curve during the baseline period. We then identified periods during the trial when (1) the curve amplitude deviated from its mean baseline activity by more than 10 SD and (2) the difference remained above two SD of the mean baseline activity for at least 125 ms. The 125-ms-duration criterion was selected because it represented about 50% of the time to reach maximal grasping forces for the two monkeys (average time between *G*_on_ and *G*_max_ = 231 ± 63 ms). A similar two-step procedure was used to compare the mean amplitude of modulation between contralateral and ipsilateral hand trials (Fig. 5*B*) and between two cortical areas (Fig. 6). Comparisons between cortical areas were done separately for each hand. In these cases, we first calculated the mean and SD of the difference between two amplitude modulation curves during the baseline period. We then identified periods during the trials when the difference between two curves was substantial using the 10 SD amplitude deviations from the baseline difference and 125-ms-duration criteria.

## Results

After cleaning the data to remove noises and artifacts (see Materials and Methods), the chronically implanted microelectrode arrays in the two monkeys provided signals from a total of 480 electrodes across different brain areas in both hemispheres. The details of data sampling from each cortical area are summarized in Table 2.

**Table 2.** Characteristics of the neural dataset

	Area	Number of recording sessions	Total number of LFP signals		Number of good signals per connector		
			Left hemisphere	Right hemisphere	Mean	Maximum	Minimum
Monkey Y	M1	6	216	–	27 ± 5	31	19
	PMd	6	230	251	25 ± 6	31	11
	PMv	6	183	277	27 ± 3	31	22
Monkey B	M1	4	189	–	21 ± 6	28	16
	PMd	5	61	105	24 ± 7	31	12
	PMv	5	80	161	24 ± 4	29	16

Data is presented as mean ± SD. Data for each area was collected over several recording sessions (Number of recording sessions). In each session, a subset of the electrodes of each implanted array were sampled. For a given area, the signals from all the good electrodes over all the sessions were pooled together (total number of LFP signals). Each array connector includes 32 electrodes or potential signals. Number of good signals per connector indicates the mean, maximum, and minimum number of signals that were kept, after excluding noisy signal.

In each recording session, a few trials (4 ± 2) per condition were removed because of artifacts (see Materials and Methods). Figure 1*B* shows the behavioral events, the time intervals between each event, and start and end times of each task epoch relative to its defining event for the two monkeys combined. Visual inspection of recorded videos confirmed both monkeys used similar kinematic strategies to perform the reach and grasp trials. The only clear difference we found was at the beginning of the trial, when the monkeys had both hands in the home plates. While both monkeys started the task from similar hand position, palms down with forearm pronated, Monkey Y tended to keep its fingers (D2–D5) half flexed while Monkey B kept fingers extended. Following the H cue, both monkeys started reaching with a slight flexion of the shoulder and the elbow. Shortly after lifting the hand from the home plate, the monkeys extended the wrist followed by a shaping of the fingers to grasp the force sensor using a vertical precision grip with the thumb and index. During grasp, both monkeys tended to slightly extend D3–D5 while they applied force on the sensor. After peak grasp force and the reward, they brought the hand back to the home plate. The details of averaged maximum force levels produced and the duration of the different phases of movement for each monkey are summarized in Table 3.

**Table 3.** Characteristics of the performed behavior

		Force (*N*)	Time between task events (ms)		
			Hand to GO	GO to *G*_on_	*G*_on_ to *G*_max_
Monkey Y	Left hand	9.4 ± 0.9	730 ± 282	456 ± 146	241 ± 62
	Right hand	11.2 ± 0.9	701 ± 279	476 ± 168	258 ± 66
Monkey B	Left hand	7 ± 0.7	639 ± 354	471 ± 132	214 ± 64
	Right hand	8.5 ± 0.7	682 ± 382	475 ± 153	209 ± 64

Data is presented as mean ± SD. Hand to GO, the interval between the hand instruction and the GO cues; GO to *G*_on_, the interval between the GO cue and the grasp onset; *G*_on_ to *G*_max_, the interval between the grasp onset and the maximal grasp.

### General pattern of LFP oscillations during contralateral hand movements

LFP spectrograms up to 60 Hz were plotted for M1, PMd, and PMv for the contralateral and ipsilateral trials for Monkey Y (Fig. 2*A*) and Monkey B (Fig. 2*B*). These spectrograms provide a broad view of the complete data set and identification of salient differences in the LFP oscillatory activity in brain areas at different frequencies for each monkey.

While there were some expected differences in spectral modulations between the two monkeys, both animals showed consistent patterns of modulations in the LFP activity in all three brain areas. Considering the overall similarity observed in the LFP activity of both monkeys, the data were pooled and averaged across animals for all analyses.

Averaged LFP spectrograms across both monkeys are plotted in Figure 3*A*. The pattern of modulation in M1 during contralateral hand trials we found was much in line with previous publications (Donoghue et al., 1998; Menzer et al., 2014; Waldert et al., 2015). There was a strong movement-related increase in the LFP power at frequencies below 13 Hz [i.e., δ (0.5–5 Hz), θ and α (5–13 Hz)] that began after the GO cue and lasted during grasp. In contrast, there was a prominent decrease of activity in the β frequency range (15–30 Hz), mostly between the GO cue and grasp onset. In the γ range (30–60 Hz), there was a moderate increase in LFP power that was aligned with the onset of grasp.

In general, the amplitude of movement-related changes in the LFP power was smaller in PMd and PMv than in M1. In PMd, much like in M1, the greatest increase of power during movements of the contralateral hand was in the lower frequencies (≤13 Hz) and peaked at grasp onset. In the β band (15–30 Hz), there was a decrease of activity early in the trial, which transitioned to an increase during grasp. In the γ band (30–60 Hz), we observed an increase of power as in M1, but that seemed less specific to any given behavioral event. In PMv, the increase of activity in low frequencies during movements of the contralateral arm was less pronounced but also peaked around grasp onset. In the β band, power was decreased throughout the trial, much like in M1. Finally, there was a slight increase of activity in the higher range of the γ frequencies we analyzed (50–60 Hz) that seemed more aligned to peak grasp force. In lower γ frequencies (30–40 Hz), there was a decrease of activity.

### Contrasting the general pattern LFP oscillations during contralateral and ipsilateral hand movements

For all three brain areas, spectral modulation of LFP during movements of the ipsilateral hand tended to follow the pattern described for the movement of the contralateral hand. However, there were also some noticeable differences. For example, the increase of activity in the lower frequencies range (≤13 Hz) in M1 was weaker during ipsilateral trials and was more centered on grasp onset. In PMd, the short increase of activity around grasp onset in the lower frequencies range was smaller during ipsilateral than contralateral grasp. In addition, there was no increase of activity in the δ range (0.5–5 Hz) during ipsilateral reaching. Of all three areas, PMv seemed to have the least pronounced differences in the pattern of modulation between ipsilateral and contralateral trials. Perhaps the most noticeable ones were in the low frequencies. During ipsilateral trials, there was a lower increase of activity around GO cue and a greater increase around grasp onset in comparison with contralateral trials.

To facilitate the visualization of effector-dependent differences in the pattern of LFP modulations, we created plots based on the subtraction of the averaged spectra obtained for the two types of trials (Fig. 3*B*; see Materials and Methods). These plots highlight that LFP modulations tended to be greater during contralateral hand movements in both M1 and PMd. In contrast, LFP power in PMv tended to be balanced during contralateral and ipsilateral trials. For both M1 and PMd, low-frequency (≤13 Hz) LFP activity was much greater during movements of the contralateral hand. In M1, this difference was particularly pronounced during reach but extended during grasp. In PMd, it was most pronounced around grasp onset. In the β range, there was a tendency for greater activity during grasp with the ipsilateral hand early in the trial and during reach in M1 and around grasp onset in PMd. In the γ range, there was a tendency for greater increase of activity during contralateral hand movements. This increased activity seemed to be time locked to grasp onset in M1 while present throughout the trial in PMd. In sharp contrast to both M1 and PMd, LFP modulations in PMv tended to be greater during ipsilateral hand movements. The most powerful modulations during ipsilateral trials occurred just after grasp onset in low frequencies and during reach in the β band.

### Contrasting the general pattern LFP oscillations across cortical areas

We also used spectrogram subtraction plots to help compare the pattern of activity in M1, PMd, and PMv (Fig. 4). Because the LFP power and amplitude of modulations were much larger in M1 than in PMd and PMv, we normalized the data within each area for each type of trials to its range of modulation (e.g., normalization within M1 for contralateral trials) before subtracting their values for comparison. This helped contrast the timing of peak LFP modulations within each area in relation to peak modulations in the other areas.

During contralateral trials (Fig. 4*A*), the plots highlight that M1 had a greater relative increase of LFP power in the low frequency range than either PMd or PMv. This was the case mostly during reach and in the δ band. In contrast, PMd had a greater relative increase of power than M1 in the β range, after maximum grasp. Comparing the two premotor areas revealed that the relative power increase in the δ and β range was greater in PMd. This was true around the onset of grasp for δ frequencies and after maximum grasp for the β range.

For ipsilateral trials (Fig. 4*B*), M1 showed a greater relative increase of LFP power than PMd and PMv in the low frequency range, during reach and early grasp. This difference was however greater for PMd than PMv. In the β range, PMd had a much greater relative increase of LFP power than M1. In comparison to contralateral movements, this difference started earlier in the trial, during reach. PMv had greater relative increase of LFP power than M1 at grasp onset in the θ and α range and during reaching and the beginning of grasp in the β range. When comparing the pattern of modulation in premotor areas, PMv had a greater relative increase during reaching in the β range and around the onset of grasp in the δ frequency range. PMd had a greater relative increase of LFP power after the maximum grasp force in the θ and α band. In the β band, the relative power increase in PMd was much greater from grasp onset to the end of the trial. Finally, PMd modulations were greater in γ frequencies during reaching.

### Quantification of LFP modulations in specific frequency bands during contralateral hand movements

For more detailed analyses, we separated the LFP activity in the δ (0.5–5 Hz), θ and α (5–13 Hz), β (15–30 Hz), and γ (30–60 Hz) frequency bands and segmented trials around task events. This increased the alignment of the LFP signals with behavior (Waldert et al., 2015; see Materials and Methods). Figure 5*A* shows frequency and event specific amplitude modulations, normalized to baseline, for contralateral and ipsilateral trials separately. All three cortical areas showed a unique pattern of modulation in each band, and this was true whether the contralateral or ipsilateral hand was used. In general, modulations of LFP power were greater in the δ and θ and α bands especially during contralateral trials. These large modulations were increases of amplitude in comparison with baseline. In the β and γ range, modulations were generally of lesser amplitude, more similar during contralateral and ipsilateral trials, and consisted of both increases and decreases of LFP power, depending on the cortical area.

We first compared the activity in each area during trials in relation to its baseline activity (Fig. 5*A*). To highlight moments with substantial changes, we used ANOVAs comparing the average activity during each epoch and a two-step approach that considered both the time and duration of modulations (see Materials and Methods). During contralateral trials, the greatest changes of LFP power in M1 were in the δ and θ and α bands. In the δ band, there was a substantial increase of power early in the trial after the hand cue that peaked during reach. δ power remained very high until the monkeys applied the maximal grasp force and returned toward baseline after maximal grasp. There was also an increase of power in θ and α range. However, it only began during reach and ended around maximal grasping force. In the β band, LFP power in M1 started to be lower than baseline early in the trial, prior to the GO cue. It remained lower until the monkeys applied the maximal grasp force and then returned to baseline by the end of grasp. Finally, in the γ frequency band, there was a short increase of LFP power at grasp onset, but that did not meet our quantitative criteria since the deviation from baseline was below 10 SD.

In PMd, there were multiple substantial modulations in the δ range during contralateral trials. There was an initial increase that occurred rapidly after the hand cue and a second during reach that peaked at the onset of grasp. Modulations in the θ and α band followed a similar pattern, although the only substantial modulation was the increase of power around grasp onset. In the β band, there was a slow increase of activity that became different from baseline after the maximal grasp force and that continued until the end of the trial. The pattern of modulations was similar in the γ band, but these changes did not meet our quantitative criteria as the deviation from baseline was below 10 SD.

In PMv, the greatest modulations in the δ band were observed at the beginning of the trial, after the contralateral hand cue, and then the activity returned to baseline during reach. The pattern of modulation was similar in the θ and α range, although only the rapid increase of power after the hand cue passed our quantitative criteria. The activity in the β and γ frequency ranges was mainly decreased during contralateral hand movements. In the β range, the decrease became substantial shortly after the hand cue and remained so into the grasp epoch. In the γ range, it started around the same time, but the activity returned to baseline values shortly after grasp onset.

### Contrasting frequencies and events specific differences between trials with the contralateral and ipsilateral hand

During movement of the ipsilateral hand, in M1 there was an increase of LFP power in the δ range that peaked during reach and came back to baseline by the end of grasp. In contrast, there were no substantial modulations in the θ and α frequency range. In the β band, LFP power was substantially decreased. This decrease started before the GO cue and reached its lowest point just prior to grasp. β LFP power then slowly returned toward baseline, while remaining lower until the end of the trial. In the γ band, there was also a decrease of power that became substantial around grasp onset.

In PMd, there were two substantial increases of LFP power in the δ range during ipsilateral trials. The first one occurred after the hand cue and a second one around maximal grasp force. LFP power then decreased back to baseline. There were no substantial modulations in other frequency bands.

In PMv, the pattern of modulation was similar in the δ and θ and α bands. There was a first substantial increase of LFP power after the hand cue and a second one that started at the onset of grasp. In the δ band, this second modulation peak lasted until maximal grasp force. In the β band, LFP power started to be decreased prior to the GO cue, until after the monkeys applied the maximal grasp force. The values returned to baseline after maximal grasp. There were no substantial modulations in the β band.

To help identify moments during trials when the difference between contralateral and ipsilateral trials was most pronounced, we made frequency-specific subtractions plots around each task event (Fig. 5*B*). We only found periods during which LFP power was substantially greater during contralateral hand movements. For all three areas, these periods were mostly present in lower frequency bands. M1 had the greatest number of different time periods across bands. In the δ band, LFP power of contralateral trials started to be greater prior to the GO cue. This difference increased during reach and peaked prior to grasp onset. It then gradually decreased but remained different until the end of the trial. The θ and α band showed a similar pattern. However, the contralateral and ipsilateral trials were not different toward the end of grasp. In M1, γ power in contralateral trials started to be greater than in ipsilateral trials at the end of reach. This difference peaked around grasp onset and rapidly decreased but remained elevated throughout the maximal grasp epoch. In PMd, LFP power was greater with contralateral hand movements only in the δ range. Much like in M1, the LFP power during the contralateral trials became substantially greater prior to the GO cue, increased during reach and peaked at grasp onset. The difference between contralateral and ipsilateral trials then decreased but remained different. In PMv, LFP power was greater with contralateral hand movements in the δ and θ and α frequencies. This difference occurred early in the trial, around the hand cue in both bands. The β band showed no period of difference between the contralateral and ipsilateral trials in any of the three brain areas.

### Contrasting frequencies and events specific LFP differences between cortical areas

A last set of subtraction plots was used to highlight differences across cortical areas in different frequency bands around each behavioral event (Fig. 6). For these plots, we calculated the LFP oscillatory activity normalized difference within each frequency band (see Materials and Methods) to help highlight the contrast in amplitude modulations between two given cortical areas. For contralateral trials (Fig. 6*A*), the activity was similar in M1 and PMd early in the trial, around the hand cue, for all frequency bands. However, in the δ range, M1 activity became greater than PMd prior to the GO cue. The difference between the two areas peaked during reach. It then decreased during grasp but remained substantially different until the end of the trial. In the θ and α band, M1 also became more active before the GO cue, but that difference only lasted until the maximal grasp force. In sharp contrast, in the β band, M1 was substantially less modulated than PMd starting before the GO cue until the end of the trial. Finally, in the γ range, M1 was more modulated for a short period, at the onset of grasp.

When comparing the activity of M1 and PMv during contralateral trials, the most striking difference was observed around the hand cue. While M1 and PMd showed similar patterns around the hand cue, the activity in PMv was greater than M1 in both the δ and the θ and α bands. By the time of the GO cue, the difference was reversed and M1 became more modulated than PMv. The pattern in these two bands then followed the one described for M1–PMd. In the β frequencies, the activity in PMv was greater than in M1, but for only a short period around grasp onset. Finally, in the γ band, the activity in M1 was slightly but substantially greater than in PMv from after the hand cue to the end of the trial.

Differences in the pattern of modulation between PMd and PMv during contralateral trials were generally of lesser amplitudes. Yet, in each frequency band, there was at least one period in the trial during which these differences were substantial according to our quantitative criteria. In the δ and θ and α ranges, PMv activity was greater around the hand cue. The δ activity then became greater in PMd than in PMv during reach and until grasp onset. In the β band, PMd was more active than PMv, starting just prior to the maximal grasp. Finally in the γ range, PMd was more active than PMv starting before the GO cue.

Differences in the pattern of modulations between cortical areas tended to be of lesser amplitude during ipsilateral trials (Fig. 6*B*). M1 and PMd had similar patterns of activity from the beginning of the trial until the GO cue. In the δ band, M1 was substantially more active than PMd from the GO cue until the onset of grasp. In the β band, PMd was more active from the start of reach until the end of the trial. There were no differences in the θ and α and the γ frequency bands.

For trials with the ipsilateral hand, M1 showed a stronger modulation than PMv from the GO cue until the onset of grasp in the δ band. PMv was more active than M1 in the θ and α band during two short periods, right after the hand cue and near the onset of grasp. In the β band, PMv was more active than M1 from the GO cue until the end of the trial. Both areas had similar patterns of activity in the γ band.

Finally, the patterns in PMd and PMv were similar from the start of the trial until grasp onset. At that point, in the δ band, PMv was substantially more active than PMd. Then, near the time of maximal grasp force, PMd became more active for a brief period followed by another increase of activity in PMv for the remainder of the trial. In the β band, PMd was more active from the time of maximal grasp. In the γ band, PMd was slightly but substantially more active than PMv for a short period around the maximal grasp force. There was no difference of modulation between the two areas in the θ and α band.

### Summary of strongest synaptic inputs modulation across M1, PMd, and PMv during reaching and grasping movements

To summarize the pattern of modulation across areas, we used the area under the curves presented in Figure 5 to identify the cortical area exhibiting the most substantial change in synaptic inputs for each frequency band during contralateral and ipsilateral trials. At the beginning of contralateral trials around the time of the hand cue, the most prominent increase of synaptic inputs took place in PMv in low-frequency bands (δ and θ and α bands). PMv could then shape M1 activity through its powerful cortical projections (Cerri et al., 2003; Schmidlin et al., 2008; Quessy et al., 2016). Afterward, the greatest increase of inputs in these bands was in M1 and started earlier in the δ than in θ and α band. Simultaneously, the most prominent modulation in both the β and γ bands were decreases of synaptic inputs. In the β band, these changes were in M1 and lasted until the end of the trial. In the γ band, they were in PMv and lasted until the onset of grasp.

For ipsilateral trials, the most prominent increase of synaptic inputs in low-frequency bands (δ and θ and α bands) first took place in premotor areas. In the δ band, similar increases were observed in both PMv and PMd after the hand cue. At the same time, PMv had strong synaptic input increases in the θ and α band. Then, after the GO cue, M1 exhibited a strong increase in the δ band, which lasted until the end of the trial. In the θ and α, PMv had a second brief increase around the onset of grasp.

Like in the contralateral trials, the greatest modulation in the β and γ frequencies were decreases of synaptic inputs. In both bands, the brain area with the most prominent decrease of synaptic inputs was M1. In the β band, it started prior to GO and lasted until the end of the trial. In the γ band, it occurred around grasp onset.

## Discussion

We compared LFP signals simultaneously recorded from M1, PMd, and PMv when monkeys performed unilateral reach and grasp with the contralateral or ipsilateral hand. The results reveal that a complex and unique pattern of modulations occurs in each area and varies in function of the hand used. We found that modulation in M1 had a greater preference for contralateral movements than premotor areas. This finding is consistent with differences observed when comparing LFPs recorded from M1 and SMA (Donchin et al., 2001). In PMd, much like in M1, there was a greater increase of low-frequency activity (≤13 Hz) during contralateral movements in comparison with ipsilateral movements. Moreover, PMd was the only area with increased modulations in the β range. PMv, in contrast, was the area with the greatest increase of low-frequency activity at the hand cue, when the monkey receives the trial instruction. It was also the area with the lowest effector-dependent modulation during movement execution. The fact that PMv receives strong inputs for movements of either hand is not surprising since studies show that most neurons in that area have similar activity for movements of both hands (Moreau-Debord et al., 2021) and are less selective for arm use than PMd (Hoshi and Tanji, 2002). Differences between M1 and premotor areas were generally greater during contralateral movements and tended to be greater than the ones between PMd and PMv. Differences between the two premotor areas tended to be greater during contralateral trials, with PMv being more modulated around hand cue and PMd during movements.

### The pattern of oscillatory activity in M1 during contralateral and ipsilateral arm and hand movements

Several anatomical and electrophysiological studies support the idea that M1 is the most lateralized cortical motor area. M1 is the primary origin of corticospinal projections, ∼90% crossing the midline to control the contralateral arm musculature (Dum and Strick, 1996; Rosenzweig et al., 2009). In M1, neurons with a preference for contralateral arm movements are more common and have stronger bias than in premotor areas (Cisek et al., 2003; Kurata, 2007). In agreement with previous LFP studies (de Oliveira et al., 2001; Donchin et al., 2001; Ganguly et al., 2009), we found that M1 had a clear preference for contralateral movements that started before movement onset and continued through reaching and grasping. This contralateral preference was most pronounced at frequencies below 13 Hz, more so in the δ band. LFP modulations likely reflect the regional synaptic inputs that drive local neural activity (Logothetis, 2002; Herreras, 2016). In this case, they could be generated by the numerous cortical inputs from premotor and somatosensory areas (Dea et al., 2016; Hamadjida et al., 2016). As these inputs come together in M1, they can shape discharges of neurons that generate corticospinal outputs and control contralateral reaching and grasping movements.

Neuronal recording experiments in M1 have also shown that the spiking activity can be related to ipsilateral proximal movements (Ames and Churchland, 2019; Cross et al., 2020). Here, we found that M1 LFPs, particularly in the δ range, are also modulated during ipsilateral distal movements. This increased δ activity occurred later in the ipsilateral trials than the contralateral trials, which suggests that ipsilateral activity may be less involved in the initiation of movements but could rather be related to some monitoring of performance. Synaptic inputs to the ipsilateral M1 could carry feedforward information about the effector available if unexpected bilateral movements need to be rapidly produced, for example, in the case of an error (e.g., drop of the object intended to grasp). Alternatively, they could reflect activity necessary to repress bilateral actions, for example, through synaptic inputs to suppression-type M1 mirror neurons (Vigneswaran et al., 2013).

M1 was also the only area that showed an increase of γ power aligned with the onset of contralateral grasp. This elevated high frequencies LFP activity during grasp could be associated with elevated spiking activity within M1 at this period (Donoghue et al., 1998; Spinks et al., 2008). Interestingly, around this time in the trial, there was a simultaneous decrease of γ rhythm in the other hemisphere. One possibility is that interhemispheric interactions between the two M1 result in a net suppression of activity in the hemisphere ipsilateral to the moving hand that favors unilateral precision grasp (Ferbert et al., 1992).

In comparison to premotor areas, the hand representation in M1, as defined using ICMS, is much larger (Dum and Strick, 2005; Dancause et al., 2006b; Boudrias et al., 2010; Dea et al., 2016). One possibility is that our sampling in M1 comprised more cortical territory exclusively involved in grasping and premotor cortex comprised more territory involved in both reaching and grasping. While we cannot completely discard this possibility, our physiological data on the location of the arrays suggest that sampling in M1 did include cortical territory involved in both reaching and grasping. This is not so surprising when considering that in macaques most of the cortex projecting to digit muscles is located deep within the sulcus (Park et al., 2001, 2004). Sampling from the convexity of central sulcus using arrays with relatively short electrodes, as done in the present study, is likely to include cortical sites with projections to more proximal muscles.

### The pattern of oscillatory activity in PMd during contralateral and ipsilateral arm and hand movements

In PMd, there was an increase of low-frequency LFP activity during execution of contralateral movements like the one observed in M1. However, there was no effector preference in the θ and α range, and this low-frequency modulation occurred later in PMd than M1. These differences between M1 and PMd are along the lines of what has been reported during movement preparation in response to reach direction cues (O’Leary and Hatsopoulos, 2006). In this context, it was found that in contrast to M1, PMd is modulated similarly in response to the instructions to either contralateral or ipsilateral trials, and that modulations in PMd occurs later than that in M1. The more similar pattern of activation we found in PMd during the execution of movements with either hand suggests that it plays a greater role in processing task requirements that are more effector-independent and to a lesser extent involved in the direct control of neurons involved in production of motor outputs to the moving hand. The early activation of PMd during instruction cues for both ipsilateral and contralateral hand trials suggests its involvement in movement preparation and is consistent with results from neuronal spiking activity studies (Hoshi and Tanji, 2002). If this information is subsequently passed along to M1, possibly through corticocortical connections (Dum and Strick, 2005; Dea et al., 2016), it could contribute to the increased low-frequency activity that starts around the GO signal in this area. After the GO signal, the increased modulations in PMd can reflect synaptic processing of inputs coming from M1. These reciprocal projections (Hamadjida et al., 2016) may serve to update PMd on the ongoing movement (O’Leary and Hatsopoulos, 2006), suggesting a dynamic interaction between M1 and PMd during the execution phase. Though most studies agreed that β power decreases in M1 and PMv following task initiation, results have been mixed for PMd (Donoghue et al., 1998; O’Leary and Hatsopoulos, 2006; Sayegh et al., 2013). In the present study, we found a substantial increase in β frequencies in PMd during the execution of contralateral grasp. Whereas there is no consensus about the functions of β activity, several studies suggested that it might be related to sensorimotor communication and reflect the information flow of inputs from the periphery to the sensorimotor cortex and other brain areas involved in sensorimotor integration (Murthy and Fetz, 1996; Kilavik et al., 2013). β oscillations could also represent a link with neural correlates of cognitive processes such as attention and rule integration (Murthy and Fetz, 1996; Buschman et al., 2012; Gallego-Carracedo et al., 2022). Thus, increased β power in PMd during grasp could highlight a unique role in the integration of somatosensory information to monitor performance during the task, in our case that appropriate forces are applied.

The increased β activity could also be caused by synaptic inputs from other higher-order cortical areas. For example, PMd receives numerous projections from the caudal part of the superior parietal lobule, area PEc (Johnson et al., 1996). PEc is a multimodal area that hosts somatosensory, visual, and bimodal neurons sensitive to eye–hand coordination (Breveglieri et al., 2008; Hadjidimitrakis et al., 2015). Information from PEc could be integrated in PMd during visually guided grasp to update PMd on grasp performance. Along these lines, enhanced β LFP power in PMd was previously reported when monkeys performed congruent eye–hand reaching movements as compared with when vision of the limb was blocked and instead the movement performance was presented on a vertical screen (Sayegh et al., 2013). In our experiments, while β oscillations were only substantially increased from baseline during contralateral grasp, we found no difference between contralateral and ipsilateral trials. This suggests that PMd may integrate sensory feedback information about both arms.

Direct comparison of LFP modulations in PMd with PMv revealed that PMd has a greater increase of synaptic inputs during contralateral movements. Interestingly, this was the case during both reaching and grasping. PMv and PMd both have neurons that discharge during grasping (Raos et al., 2004, 2006). Recent studies directly comparing neuronal activity in PMd and PMv have also reported modulations in the two areas in both phases of movements (Takahashi et al., 2017; Atique and Francis, 2021). The involvement of PMd in grasping can be supported by its numerous cortical connections with the hand representation in M1 (Stepniewska et al., 1993; Dum and Strick, 2005; Dea et al., 2016; Hamadjida et al., 2016) and its corticospinal projections to lower cervical segments, which are much more numerous than the ones from PMv (He et al., 1993).

### The pattern of oscillatory activity in PMv during contralateral and ipsilateral arm and hand movements

Overall, PMv showed the least bias for contralateral trials and only had substantial grasp-related modulations in low frequencies during ipsilateral trials. The observed low effector-dependent LFP activity in PMv aligns with findings from studies on its spiking activity. Previous studies have found that many PMv neurons show little selectivity for the hand used but are rather coding for the location of the target, its shape, or the configuration of the hand to grasp that target (Tanji et al., 1988; Rizzolatti and Luppino, 2001; Michaels and Scherberger, 2018). Using cortical array recordings as in the present study, we also found that PMv neurons have a low effector-dependent preference (Moreau-Debord et al., 2021). The lower effector-dependent LFP activity in premotor areas in comparison with M1 could be attributed to the fact that these areas receive inputs to build “higher-level” components of movements such as the hand configuration necessary for grasping an object. The specialized aspects of the higher-level constructs carried by each premotor area are likely determined by its unique input pattern (Ghosh and Gattera, 1995; Matelli et al., 1998; Tanné-Gariepy et al., 2002; Dancause et al., 2006a). Premotor areas could then send this information to the M1 contralateral to the hand that will be used, through both their numerous intra and interhemispheric projections (Rouiller et al., 1994; Marconi et al., 2003; Boussaoud et al., 2005; Dancause et al., 2007).

Much like in M1, we observed a decrease of β oscillations in PMv during reach and grasp, with no difference in relation to the laterality of the effector. β modulations in PMv contain grasp-related information and are present, although to a lesser degree, during movement observation (Waldert et al., 2015). However, the greatest modulations in PMv were observed in δ and θ and α bands during trial instructions. These findings are in agreement with earlier grasp-related modulation of spiking activity seen in PMv neurons (Umilta et al., 2007). This early low-frequency burst suggests that PMv may receive stronger and earlier synaptic inputs about visual cues than PMd and M1. It may reflect the importance of PMv in early processing for action planning and transferring of visuomotor information into motor commands (Umilta et al., 2007; Davare et al., 2009). In the hemisphere contralateral to the effector, this information can then be used to increase M1 excitability and shape its outputs, for example, through the cortical projections of PMv to M1 (Shimazu et al., 2004; Dancause et al., 2006a; Schmidlin et al., 2008).

There was also an increase of PMv activity in low frequencies while monkeys were grasping (Figs. 2, 3*A*), but these modulations were only substantial during ipsilateral trials (Fig. 5*A*). Increased activity in low frequencies during grasp may represent synaptic inputs updating PMv on movement performance. These LFP modulations suggest that PMv in both hemispheres could be concurrently contributing to grasp. Invasive stimulation studies have shown that PMv exerts strong facilitation on M1 within the same hemisphere (Cerri et al., 2003; Shimazu et al., 2004) but has predominant inhibitory effects on the opposite M1 (Quessy et al., 2016). One possibility is that while PMv in the hemisphere contralateral to the hand facilitates M1 neurons to produce the motor outputs, the ipsilateral PMv could simultaneously inhibit other neuronal populations through its callosal projections (Boussaoud et al., 2005; Dancause et al., 2007) to prevent undesirable cocontractions and refine the output pattern.

We found that the modulation in the γ band in PMv was decreased prior to the GO signal and around the onset of grasp. These changes could reflect a decrease of interaction between GABAergic interneurons and pyramidal cells in PMv (Buzsaki and Wang, 2012). Decreased γ activity could be considered somewhat surprising given that neuronal activity takes place in PMv during both contralateral and ipsilateral grasping movements (Tanji et al., 1988; Hoshi and Tanji, 2002; Moreau-Debord et al., 2021). Along these lines, another study on LFP recordings in macaques during grasping has documented an increase of activity in PMv in the γ range, specifically above 50 Hz and only in one animal (Waldert et al., 2015). Interestingly, in both of our animals, we also saw an increase of γ activity at frequencies above 60 Hz peaking around maximal grasp force (data not shown). This divergent pattern in low and high γ frequencies was more obvious in PMv than in PMd or M1. In macaque monkeys, others have defined the range of β oscillations between 25 and 40 Hz (Khanna and Carmena, 2017). One possibility is that the power attenuation we observed and report as being in the low γ range (30–40 Hz) is in fact part of the expected decrease in β power. Alternatively, our results could suggest the possibility that there are two concurrent patterns of activity occurring in the γ band, a decrease in low γ range (30–50 Hz), and an increase at higher frequencies (>50 Hz). Different patterns of activity and potential functions across distinct narrow bands of γ frequencies have been proposed, for example, in the visual and sensorimotor cortex (Crone et al., 1998; Han et al., 2021). In the visual cortex, it was suggested that low and medium γ frequencies are primarily caused by cortical excitatory and inhibitory interactions and high γ frequencies by inputs from subcortical regions (Han et al., 2021). Similarly, low and high γ modulations in PMv could be related to different networks and processes that take place during the generation of grasp.
